# A Qualitative Study Exploring Diabetes Prevention Intervention Needs and Ideas Among Young Adults at High Risk for Diabetes

**DOI:** 10.1016/j.jneb.2026.01.014

**Published:** 2026-02-18

**Authors:** Abigail Arons, Mai Baalbaki, Susan D. Brown, Amy L. Beck

**Affiliations:** 1 Division of General Internal Medicine and Bioethics, Department of Internal Medicine, University of California Davis, Sacramento, CA; 2 Institute for Health Policy Studies, University of California San Francisco, San Francisco, CA; 3 Division of General Pediatrics, Department of Pediatrics, University of California San Francisco, San Francisco, CA

**Keywords:** young adult, prediabetes, diabetes prevention, tailored intervention

## Abstract

**Objective:**

To explore perspectives of young adults with prediabetes and high burden of social needs about their intervention needs and ideas for solutions to enable behavior change.

**Design:**

Qualitative study with semistructured interviews.

**Setting:**

One safety-net hospital and 1 academic medical center in a US city.

**Participants:**

Twenty-four publicly-insured adults aged 18–26 years with prediabetes (63% female, 71% Latino/a/x, 29% Black, 61% food insecure, and 39% any college; mean age 22.6 years).

**Phenomenon of Interest and Variables:**

Internal and external factors affecting dietary and exercise behaviors, within the Capability-Opportunity-Motivation-Behavior framework.

**Analysis:**

Data were analyzed using a general inductive approach followed by directed content analysis within the Capability-Opportunity-Motivation-Behavior framework.

**Results:**

Participants reported few intervention needs regarding Capability, with sophisticated nutrition knowledge and an accurate understanding of prediabetes. Major Opportunity needs emerged, including the high cost of food not offset by the food safety net, internalized effects of immersion in unhealthy environments, and low control over time. Motivation varied: most desired to avoid diabetes, but interviewees had mixed perceptions of their risk and self-efficacy. Interviewees shared 23 Intervention Ideas to address identified needs.

**Conclusions:**

Our findings can inform youth-centered multimodal diabetes prevention interventions for young adults at high risk of diabetes that leverage existing knowledge, address Opportunity barriers, and differentiate among subgroups with varying motivation.

## INTRODUCTION

Young adulthood is an important time for diabetes prevention,^1,2^ yet current interventions do not meet young adults’ needs. In the US, 24% of adults aged 19–34 years have prediabetes.^3^ This age is a developmental period that has a long-lasting impact on the future risk of diabetes. For example, weight gained during young adulthood carries a higher risk of diabetes compared with weight gained at older ages,^4,5^ and lasting dietary and physical activity behaviors are established in young adulthood.^6,7^ While lifestyle interventions are first-line therapy for reducing diabetes risk in other age groups, young adults benefit less than their older counterparts across clinical trials and observational studies.^8–10^ This discrepancy is likely multifactorial. Young adults may not have the health literacy to gain access to lifestyle interventions or to clinical trial participation,^11,12^ and young adults have higher levels of uninsurance than other age groups.^13^ Given evidence of disparities in clinical trial enrollment^14^ and access to lifestyle interventions,^15^ this is likely especially true for young adults from marginalized races and ethnicities, and lower socioeconomic status, who are at higher risk of diabetes. Even when enrolled in lifestyle interventions, young adults experience lower engagement than their older counterparts.^8,16^ This suggests a need for tailored diabetes prevention interventions for young adults. Despite young adults’ distinct intervention needs, there have been 2 pilot studies^17,18^ and no large clinical trials of tailored diabetes prevention interventions for young adults.

Given well-documented challenges in engaging young adults in interventions, garnering their perspectives on intervention needs and ideas early in the design process is a best practice that may improve intervention effectiveness.^19,20^ Existing literature provides initial insights into young adults’ intervention needs, including survey studies showing that young adults have mixed accuracy in their perceptions of diabetes risk.^21–26^ Studies of multimodal obesity interventions for young adults have had variable efficacy, with engagement identified as a key challenge.^27,28^ These young adult-tailored studies are not specifically tied to diabetes prevention, and their study samples include primarily White, higher socioeconomic status (SES) populations.^21–27,29^ Tailoring preventive interventions to young adults from minoritized races and ethnicities and lower SES is especially important given strong evidence that external environmental and social factors, such as neighborhood deprivation and chronic stress, are drivers of diabetes risk.^30,31^ Young adults facing such external influences likely have distinct intervention needs from others in their age group.

Qualitative studies can elicit and elevate perspectives from marginalized communities that may be overlooked in large-scale studies and generate patient-centered hypotheses to be tested in future work.^32^ A few researchers have previously used qualitative methods with young adults at-risk for diabetes from low SES groups to better understand factors driving lifestyle behaviors. One interviewed Latino/a/x young adults, but exclusively focused on the role of family context.^33^ Another study conducted focus groups with Black young adults to describe their approaches to health decision-making through a behavioral economics lens.^34^

Building on this work, our objective was to directly elicit a fuller picture of intervention needs from the perspective of young adults at-risk for diabetes (with prediabetes and from low SES backgrounds), and their ideas for interventions to address these needs. The goal of this work was to round out the exploratory phase of intervention design and inform the development of tailored young adult diabetes prevention interventions for high-risk populations.

## METHODS

### Conceptual Model

Our approach was structured around the Capability Opportunity Motivation-Behavior (COM-B) model of health behavior change.^35^ The COM-B model has been widely applied to inform intervention development, including within the Consolidated Framework for Implementation Research.^36^ We elicited participants’ perspectives on intervention needs and their ideas to meet those needs within each of COM-B’s 3 domains: Capability (knowledge and skills to carry out a behavior), Opportunity (external physical and social contexts that influence behavior), and Motivation (automatic and reflective behavioral drivers). We centered on participants’ real-life experiences with existing resources and interventions (e.g., school-based activities, conversations with providers, food safety-net programs) to ground the conversations. This basic qualitative study was structured around an interpretive study design.^37^

### Recruitment and Eligibility

We recruited adults aged 18–26 years from 1 safety-net hospital and 1 academic medical center in a single urban US city. Eligibility criteria were: having prediabetes (hemoglobin A1c 5.7% through 6.4% or chart diagnosis—an indicator of increased risk of developing future diabetes^38^) in the past 4 years (to account for loss to follow-up in pediatric to adult transitions), awareness of one’s elevated diabetes risk (given ethical concern with disclosing a new diagnosis of prediabetes via research), ability to participate in an interview in English or Spanish, and Medicaid at the most recent visit (Supplementary Table 1). Patients with diabetes were excluded.

We identified patients via electronic health record (EHR) query, then randomly ordered them within 4 groups by language and gender (Spanish-speaking female, Spanish-speaking male, English-speaking female, English-speaking male). We issued invitations within each group until thematic saturation was reached or all potentially eligible patients in the group had been contacted. We mailed invitations via letter, with follow-up by phone calls and text messages.

Participants gave verbal consent at the time of scheduling their interview. Per institutional policy, primary care providers could opt out patients before invitation. The Institutional Review Board at the University of California San Francisco granted approval via expedited review for the study.

### Study Procedures

Two researchers conducted virtual, one-on-one, 1-hour semistructured interviews from January to September 2024, in the language of subjects’ choice (English or Spanish). One interviewer had prior experience, and the other was trained by observing several interviews before interviewing independently. The bilingual principal investigator conducted all Spanish interviews. All interviews were recorded and professionally transcribed in their original language. The research team developed the interview guide (Supplementary Table 2) with input from experts in young adult behavior change and diabetes prevention, and mock-tested it with a qualitative researcher outside the research team. The guide had 5–10 questions in each of 4 sections (lifestyle and knowledge background, opinions on challenges/facilitators to lifestyle change, diabetes awareness, and Intervention Ideas) and was designed to take 45–60 minutes to complete.

To gather background demographic information on interviewees, all subjects completed a 33-item online survey via Research Electronic Data Capture in English or Spanish before their interviews. Survey questions included: age, race (self-reported checking all that applied from a list of options including Black or African American, Asian, Native Hawaiian or other Pacific Islander, American Indian or Alaska Native or Other), ethnicity (a yes or no response to are you Hispanic or Latino[a]), primary language, education, income, living arrangement, food insecurity (Hunger Vital Sign^39^), food program participation (*Supplemental Nutrition Assistance Program* [SNAP], *Special Supplemental Nutrition Program for Women Infants and Children*, food bank, campus food pantry, other), self-reported health status (poor/fair/good/excellent), family history of diabetes, and comorbidities (checklist of conditions).

### Analysis

To analyze interview transcripts, we first used a general inductive approach^40^ to generate initial codes, followed by directed content analysis^41^ to organize and analyze these codes within the COM-B framework informed by the constant comparative method.^42^ This combined approach allowed us to remain young adult-centered by using a data-driven method to describe interviewees’ perspectives while also mapping these perspectives to an evidence-based framework to inform future intervention design.

Initial codes were developed inductively from the interview transcripts. Two authors began by reading the first 6 transcripts independently and then collaboratively created detailed codes that described the meaning of all text segments. At this stage, the team turned to directed content analysis, organizing these codes into the COM domains and a separate category for Intervention Ideas. The team determined which codes needed to be added to ensure each domain had sufficiently granular codes (i.e., we added detailed codes on aspects of Knowledge in the Capability domain). Then, using qualitative analytic software (Dedoose), 2 experienced qualitative coders independently read all English manuscripts and applied the codes to each text segment, meeting weekly to compare and discuss each transcript’s coding in detail, and reconcile conflicting codes. Together, 3 authors reviewed and updated the codebook after the first several interviews were coded, and then recoded the initial interviews. One author then coded all Spanish interviews and discussed each coding in detail with the bilingual senior investigator, then read and independently reviewed and adjusted the coding of the Spanish interviews.

Once we had coded all text segments, we compiled all excerpts within the domains of COM and Intervention Ideas and reviewed them within each domain separately to determine summative themes within that domain. We organized excerpts into examples/counterexamples within themes, and the themes were refined through iterative group discussion. Consensus on the final set of major themes and representative quotes was achieved among the research team.

After determining major themes, we performed additional focused inductive analysis in the Motivation domain, in which interviewees expressed substantially divergent views, supported by a behavioral psychology expert, who, with another author, rereviewed the full transcripts to identify patterns of participants with contrasting perceptions of diabetes risk and self-efficacy for behavioral change, along with supporting quotes, which the full research team reviewed.

Next, the authors mapped interviewees’ coded Intervention Ideas back to the intervention needs themes of the preceding COM-B analysis. Finally, we translated selected quotes from Spanish interviews to English for presentation.

We used multiple strategies to ensure the trustworthiness of the analysis.^43^ Once we had the preliminary analysis completed, we summarized themes and garnered feedback from a variety of stakeholders. This included member checking, in which we created a webpage summarizing themes that was shared with all participants with a link for feedback. We additionally invited 2 interviewees who had expressed interest in virtual meetings to discuss the results, although both ultimately declined. We engaged in peer debriefing via interactive presentations with a group of teens and young adults and separately with a group of diabetes translational researchers. We additionally fully describe the context and methodology herein.

We compiled descriptive statistics using survey responses. We assessed selection bias by comparing EHR demographic data from the original query (gender, primary language, age, months since last primary care visit) for the full contacted population vs interviewees, using chi-square and t test of independence.

## RESULTS

### Recruitment and Sample Characteristics

Of 228 potential participants contacted, we recruited 24 interviewees for an overall yield of 10.5% (Supplementary Figure). Interviewee mean age was 22.6 ± 2.4 years (range 18–26 years); 63% were female. On the basis of survey results, interviewees’ self-identified race and ethnicity were 71% Latino/a/x, 29% Black; 42% identified as primarily Spanish speaking, 43% reported having children, 39% were current/prior college students (Table 1). Compared with EHR data for all contacted patients, the final interview sample was more female (63% vs 40%, *P* = 0.04) and had a shorter duration since last primary care visit (13 months vs 21 months, *P* = 0.02). There were no statistically significant differences in language or age (Supplementary Table 3). Recruitment yield varied by demographic group (Supplementary Table 4).

### Intervention Needs by COM-B Domain

Results of the thematic analysis of intervention needs, including illustrative quotations corresponding to each COM-B construct, are presented in Tables 2 and 3.

#### Capability (knowledge and skills).

##### The gap between knowledge and behaviors.

Most participants reported sophisticated dietary knowledge, mainly from school and clinicians (e.g., nutritionist, doctors). This included attention to balancing protein and carbohydrates, using tools such as MyPlate, and a perception that processed foods, high sodium, and high sugar foods were less healthy. For instance, an interviewee described a healthy diet as “not too many carbs, not too much like sugary foods, also prioritize our water consumption as well, stay away from like the sweets, and the cakes, and juices with like high sugar.” Several endorsed intuitive eating, paying attention to their body’s needs to determine healthful intake. Almost all participants named sugar-sweetened beverages, such as soda and juice, as particularly harmful. Despite their knowledge, participants noted their actual eating behaviors were not fully consistent with what they knew to be healthy and felt they lacked skills to operationalize their knowledge (e.g., how to cook healthy foods).

Similarly, participants had an accurate understanding of prediabetes and diabetes complications. Nearly all participants understood prediabetes as a precursor to developing diabetes, which could be addressed by improving diet and exercise. As described by an interviewee, prediabetes means “at elevated risk to be one of the types of diabetes. … constantly managing to try not to actually be diagnosed with actual diabetes. So, it’s treatment and changing food habits.” Interviewees had witnessed family members with diabetes and could list potential complications, including needing to inject insulin, risks related to eyesight, kidneys, and amputations. However, most felt they were not addressing this risk in their lives, for example, “Some days I definitely look out for what I eat, and then some days I just can’t help it.”

#### Opportunity (physical and social context).

##### Healthy food remains prohibitively expensive, despite existing food programs.

Most participants considered the cost of food as a primary barrier to healthy eating, describing less healthful food as cheaper and healthy food, such as salads, as more expensive. A common sentiment was, “I think it’s more expensive to maintain a healthy diet.” Many revealed specific instances in their lives when finances were tight and they had to choose less healthy foods.

Most interviewees had personal experience with food programs, including SNAP and food pantries. Some credited these programs with improvements in their dietary quality; others saw the programs as providing necessities that may not be healthy. As an interviewee said, “I do have an EBT card. But does it make it healthier? No. I think it’s just still like an influence like what you’re already programmed to eat. What you crave.” Many reported challenges with food program access, including difficulty enrolling in and maintaining SNAP benefits and inconvenient food pantry schedules. Barriers to food program uptake across participants included a perception that others had higher needs, desire to make changes without relying on others, shame, and negative experiences (e.g., rotten food in a pantry box).

##### Transportation and child care needs restrict the ability to access resources.

Transportation and child care needs restrict the ability to access resources. Interviewees frequently cited transportation and child care as limiting their ability to participate in existing resources. This included difficulty carrying campus food pantry boxes on public transportation and needing child care to make use of a free gym downstairs. One interviewee said, “I actually have a gym membership at a nice gym. I just haven’t gone because I can’t afford gas every single day.

##### Immersion in unhealthy environments makes change challenging.

Interviewees saw themselves as immersed in physical and social environments they perceived as unhealthy, making change seem out of reach. Many explicitly described their low-income neighborhoods as having predominantly unhealthy food choices, including fast food, with few grocery stores and few safe exercise spaces. Socially, most interviewees described their own cultural foods (Hispanic and/or Black) as inherently unhealthy, and nearly all described their childhood family food environment in a negative light. For instance, an interviewee said,

I am one of those children that was brought up, a lot of dino nuggets at home or going to McDonald’s and stuff, and eating chips. So, not throwing shades at like any parents, but you know, sometimes it’s hard to get away from that once you’re older.

In addition to lasting childhood influence, many described continued situations in which family members bought and prepared unhealthy food. Several described feeling more able to enact change after moving away from home. Many saw social media as an additional harmful environment that promotes unhealthy foods, negative body image, and unrealistic lifestyles.

##### Lack of control over time hinders change efforts.

Almost all participants cited time demands as a major barrier to exercise and healthy eating. Interviewees had busy schedules with inflexible school times, shift-based employment, and caregiving responsibilities (for both their own children and other family members). As an interviewee put it:

I don’t have enough time. … I have to sacrifice one thing for another. I need school because I need it for my career, and I can’t drop out of school for my health. I know I need my health, but everything is working against me. It’s either my health or my career.

Several did not feel empowered to make time for healthy behaviors, for instance, when they were scheduled for work shifts during usual exercise times.

#### Motivation.

##### Learning of diabetes risk is a wake-up call that can be hard to sustain.

Several participants described learning of their prediabetes as a wake-up call and had strong emotional reactions, including sadness and fear, after conversations with providers. As one described, “I’m not gonna lie, it went through one ear…but when I heard prediabetes that’s where like it really hit. It hit me really hard like, ‘Okay, it’s getting serious.’” Similarly, another said, “It was kind of a wake-up call. I was like, ‘I need to eat better before I end up like my *tías* and my *tíos*.’” Immediately after a clinic visit, participants made behavior changes as recommended by providers to lower their diabetes risk, such as switching from sugar-sweetened beverages to water. However, multiple participants described stopping new healthy behaviors in the months following a visit, including if follow-up lapsed, or their hemoglobin A1c returned to normal. Interviewees who were parents themselves in particular felt strongly motivated to stay healthy for their children, but were also isolated and often unsuccessful in their attempts adopt healthier behaviors.

#### Motivation.

##### Identification of 4 subgroups with distinct needs to support motivation to make behavior changes.

Interviewees diverged in their motivation to enact behavior change, with 4 subgroups emerging that varied in their perceived personal risk of diabetes, perceived benefits of lifestyle change to reduce diabetes risk, and self-efficacy in making lifestyle change (Table 3). These subgroups included (1) an actively optimistic group with high perceived diabetes risk perception, high perceived benefit of lifestyle change, and high self-efficacy, who believed they would successfully enact behavior change and avoid diabetes; (2) a group that was worried but struggling to change with high diabetes risk perception and high perceived benefit of lifestyle change but low self-efficacy, who believed they needed to make lifestyle change to avoid diabetes but doubted their ability to do so; (3) a fatalistic group with high diabetes risk perception but low perceived benefit of change who considered diabetes as inevitable; and (4) a low risk perception group who did not think they had risk of diabetes, seeing no need for lifestyle change.

### Intervention Ideas

Interviewees shared a wide range of ideas for potential interventions to meet their identified needs within the COM domains (Table 4). Interviewees predominantly desired fun, experiential learning activities, such as field trips and hands-on cooking or exercise classes. Many ideas included the provision of resources, such as free food, child care, gym memberships, or support with benefits enrollment. Several interviewees desired coping support, via coaches, peer sharing, and mental health care. Specific suggestions to enhance young adults’ ability to engage included having flexible drop-in hours at settings in which youth already are (e.g., community college campus, neighborhood basketball court), teaching how to use social media in positive ways (e.g., finding home workouts), offering multiple scheduling options, including virtual delivery, and using texting for accountability check-ins. These ideas were primarily grounded in prior experiences with programs (not necessarily diabetes-specific), and elements interviewees would suggest either replicating or doing differently.

## DISCUSSION

We interviewed young adults with prediabetes and a higher burden of social needs than most previously studied populations, using an actionable, theory-based framework to elicit their perspectives on intervention needs and potential solutions. These rich conversations yielded numerous insights. Interviewees reported relatively sophisticated knowledge about prediabetes and dietary recommendations, and most desired to avoid diabetes, wanting more for themselves than their parents’ generation. However, they struggled to translate their knowledge and fear of diabetes into sustained action, facing difficulty overcoming both environmental and internal barriers. Interviewees shared broad-ranging ideas for increased support through multimodal intervention.

For Capability, participants’ preexisting knowledge was an asset, with interviewees identifying intervention needs for turning this knowledge into action. This included both dietary knowledge that appeared more sophisticated than in prior research,^44^ and an accurate understanding of both prediabetes and diabetes, beyond that seen in earlier survey studies,^21–26,29^ An approach that assumes fairly high baseline knowledge and focuses on knowledge translation diverges from existing interventions for both young adult obesity^28^ and the *Diabetes Prevention Program* core curriculum. Instead, study participants desired experiential opportunities to help them translate their knowledge into action (e.g., cooking and exercise classes).

In the Opportunity domain, intervention needs that participants identified—cost of food, unmet economic needs (i.e., transportation, child care), and environmental disparities in access to healthy food and exercise—are well-established determinants of lifestyle behaviors.^30,33,34^ Many interviewees elevated these as essential challenges that interventions should explicitly address, without which lifestyle change seemed impossible. This is consistent with the socioecological model, wherein community and environmental influences strongly affect health behaviors.^45^ However, few lifestyle interventions have incorporated direct support in meeting social needs.^28,46^ The limited examples mainly include food insecurity interventions,^46^ which are likely important for young adults. However, participants’ intervention needs went beyond the provision of food supports. Transportation and child care were key limiting factors that would need to be addressed to enable participation. Furthermore, our interviews revealed a complex picture of how participants internalize and respond to immersive external factors that shape their social and physical environments. Interviewees were aware of and felt limited by these factors, and desired that interventions acknowledged their reality, with some hoping to share strategies to thrive within their current environments. Elements of existing interventions (e.g., group coaching^47^) could be adapted to support this objective, and curricula reviewed by young adults with lived experience could further ensure lessons account for youth’s reality. While a few interviewees mentioned systemic policy-level solutions, such approaches could further mitigate external barriers to change.

Motivation is a focal point of lifestyle interventions. Interviewees generally corroborated that avoiding diabetes is a specific driver of motivation for young adults with prediabetes, consistent with studies in older adults.^48^ However, interviewees fell into 4 distinct subgroups expressing different perceptions of diabetes risk, benefits of lifestyle change, and self-efficacy. These dimensions of perception align with multiple relevant theories, including social cognitive theory that emphasizes self-efficacy^49^ (and strongly informs the theoretical basis of *Diabetes Prevention Program*^50^), and the Health Belief Model that emphasizes perceived susceptibility and benefits to change.^51^ However, these concepts had not previously been applied jointly to characterize young adults’ motivation in detail. Prior young adult studies^21,26^ have described groups with fatalism or low perceived risk. While some interviewees expressed these ideas, many others were instead either actively optimistic and taking steps to reduce risk, or at least considered it possible to prevent diabetes even though they struggled to take action. Delineating these subgroups is important for future intervention design, as intervention needs would likely vary depending on a person’s baseline perception of risk and self-efficacy for lifestyle change.

This study was subject to limitations. We applied an exclusion criterion for those who were not aware of their elevated diabetes risk. Only 7% were excluded for this criterion, though others may have declined responding to outreach. As with all qualitative studies, these results may not be fully transferable to other young adults. In particular, our findings may not be transferable to young adults unaware of their increased diabetes risk. Subjects were recruited from a single geographic location, likely with similar cultural, schooling, and health system experiences that may not generalize to other localities. Additional quantitative studies informed by our findings, as described in the Implications section, could help fill this gap.

## IMPLICATIONS FOR RESEARCH, PRACTICE AND POLICY

Young adulthood is a critical window for preventing diabetes, but effective preventive interventions are lacking for this age group. In our interviews with young adults with prediabetes and high social needs, participants revealed an overarching desire to reduce their future diabetes risk. However, interviewees expressed a need for tailored, multimodal strategies that leverage existing knowledge, while accounting for the complexity of external circumstances impacting young adults’ ability to make lifestyle changes.

Importantly, interviewees shared numerous concrete ideas for possible intervention components that map to identified needs. These included a desire for programming in convenient locations to young adults (community college, local park), and activities focused on fun, experiential learning. Desired lesson content included skill-building around strategies to overcome challenging environments, and skills that youth may not have learned from their parents. Most desired programs that include concrete resources, such as access to healthy food or child care. These ideas can serve as a starting menu of options for future co-design processes with young adults that can be tailored to meet local needs.

In addition, our key findings can inform future young adult-centered research questions to better understand young adult diabetes prevention needs at the population level. For example, our findings of relatively strong health knowledge point to a need for more dedicated studies of nutrition and health literacy in this age group, to understand strengths and when gaps may remain. In addition, quantitative studies can seek to disentangle the complex interaction interviewees described between food insecurity, food program support, and diet quality in the transition to adulthood, to inform more tailored food support resources during this time. Finally, our finding of specific subgroups that varied in their motivation and self-efficacy to avoid diabetes highlights a need for more nuanced, young adult-centered questions for surveys seeking to understand young adults’ diabetes prevention perceptions. Together with insights from new co-designed interventions, these research questions can help advance the field of young adult diabetes prevention, moving toward more patient-centered, effective prevention for this age group.

## Supplementary Material

Supplementary Material

Supplementary data related to this article can be found at https://doi.org/10.1016/j.jneb.2026.01.014.

## Figures and Tables

**Table 1.** Characteristics of the interview sample based on survey responses

Characteristic	Mean ± SD (Range) or n (%)
Age	22.6 ± 2.4 (18–26)
Gender	
Female	15 (63)
Male	9 (38)
Other/nonbinary	0 (0)
Race/Ethnicity (includes multiple answers)	
White	3 (13)
Black	7 (29)
Asian	1 (4)
Native Hawaiian or Pacific Islander	1 (4)
American Indian or Alaska Native	2 (8)
Latino/a/x	17 (71)
Primary language spoken at home^a^	
English	14 (58)
Spanish	10 (42)
Other	0 (0)
Born outside US	6 (25)
Have children/currently expecting	10 (43)
Live with parents	10 (43)
Any postsecondary education	9 (39)
Food security	
Food insecure (Hunger Vital Sign)	14 (61)
On SNAP and/or WIC	14 (61)
Food insecure and not using food programs	6 (26)
Health	
Self-rated health good or very good	13 (56)
Family history of diabetes	16 (70)
Physical health comorbidity	11 (48)
Mental health comorbidity	8 (35)
Any physical or mental health comorbidity	14 (61)
Ever attempted lifestyle changes to be healthier before	23 (100)

EHR indicates electronic health record; SNAP, Supplemental Nutrition Assistance Program; WIC, Special Supplemental Nutrition Program for Women, Infants, and Children.

a All participants were given a choice of English or Spanish for their interview and survey, regardless of the language listed in EHR; n = 5 interviews were conducted in Spanish, and n = 19 were conducted in English.

Note: The number of participants for age, gender, race/ethnicity, and primary language was 24, whereas for all other results, as one participant did not complete the survey, was 23.

**Table 2.** Selected Quotes Related to Capability and Opportunity for Diabetes Prevention and Lifestyle Change

Theme	Example Quotes
Capability	
Relatively strong knowledge of nutrition	E24: …[in school] they would teach us about the food groups. They would teach us the plate; what would you put on your plate? Like how much fruit or vegetables, grains, proteins you would eat…In high school we started learning about what’s in our soda and what was actually in the Coca-Cola soda bottles. E14…not too many carbs, not too much like sugary foods, also prioritize our water consumption as well, stay away from like the sweets, and the cakes, and juices with like high sugar, and stuff like that, or like sodas.
Accurate understanding of prediabetes and diabetes complications	E18: Once your AC1 levels reach, I believe, 5.9 or above, you’re almost like prediabetes. And you’re just a couple numbers away from having diabetes. S8: Prediabetes goes in phases. I think this is the first stage, before you have diabetes. E3: at elevated risk to be one of the types of diabetes. …constantly managing to try not to actually be diagnosed with actual diabetes. So, it’s treatment and changing food habits. S10 I know that people who suffer from diabetes, then a little wound, anything, they can suffer from some infection, lose limbs and things like that, that is, their life is more delicate.
Behavior not consistent with knowledge	E6: I’m definitely undisciplined when it comes to being strict on my diet… Some days I definitely look out for what I eat, and then some days I just can’t help it. S10: I had a nutritionist at my clinic, who even gave me vouchers to buy healthy food… I feel like it took a long time, and I wasn’t able to learn and grasp, so to speak, my habits and discipline again… That’s what I need to learn. Not to know the same thing about vegetables.
Opportunity	
Healthy food prohibitively expensive, despite existing food programs	E3: Some of us are in debt or going to be in debt or we’re on a tight budget. And it’s like, “Oh, buying a salad is $10, or like 15, 20.” And buying a burger is way less. S9: I think it’s more expensive to maintain a healthy diet. It’s more expensive E24: I do have an EBT card. But does it make it healthier? No. I think it’s just still like an influence like what you’re already programmed to eat. What you crave.
Transportation and child care needs restrict ability to access resources	E21: I actually have a gym membership at a nice gym. I just haven’t gone because I can’t afford gas every single day. E5: my best friend, she’s been telling me for almost two months to go to the gym with her and everything, to leave my kids in the night with my baby daddy, and to go.
Immersion in unhealthy environments makes change challenging	E20: certain stores and certain neighborhoods they don’t really supply healthy foods E16: I am one of those children that was brought up, a lot of dino nuggets at home or going to McDonald’s and stuff, and eating chips. So, not throwing shades at like any parents, but you know, sometimes it’s hard to get away from that once you’re older. E14: before I moved out and I met my husband, I lived in a Hispanic household. So, we eat like a lot of bread, and soda, and stuff like that. So, after I moved out, I had a chance to just purchase different stuff that I wanted to eat, and I had more control over it rather than having it in front of me and just indulge in it.
Lack of control over time makes change overwhelming	S9: My time is divided every day into the same routine between being a mother, being a housewife and being a worker. So, even if I wanted to, I don’t see the time to be able to dedicate myself to exercising. E12: I started working in the morning and that was the time I would go to the gym and I really didn’t wanna go after work ’cause I’m already tired from work. S10: I don’t have enough time. Right now, I have to talk to you because there wasn’t enough time. I didn’t go to class, and I mean, I have to sacrifice one thing for another. I need school because I need it for my career, and I can’t drop out of school for my health. I know I need my health, but everything is working against me. It’s either my health or my career.

**Table 3.** Four Subgroups With Varying Motivation to Engage in Lifestyle Change

Group	1. Actively Optimistic	2. Worried But Struggling to Act	3. Fatalistic	4. Low Perceived Risk of Diabetes
Perceived personal risk of diabetes	High	High	High	Low
Perceived benefits of lifestyle change	High	High	Low	−
Self-efficacy in making lifestyle changes	High	Low	−	−
Example quotes	E18: I had that feeling [that I would get diabetes] before I started losing weight. Now that my weight is significantly less, also getting my A1Cs checked pretty often, so just making sure I’m not in that range E6: [the thought of diabetes] just makes me wanna eat healthier. Just makes me wanna do more exercise. Just do the best effort I can to not be at risk and not fall. ’Cause at the end of the day…I do have a choice whether to eat bad or not.	E3: I feel like [diabetes] is probably not something that I would like to happen. I feel like a lot of lifestyle changes that I have to make… I’m constantly trying to lose weight, but it’s kind of hard.” S7: [Diabetes] scares me a lot…honestly I haven’t [made lifestyle changes]… it’s very complicated for me.. I say today I’ll eat better and tomorrow I don’t, and tomorrow goes by and I keep not doing it.	E1: I wouldn’t be upset, honestly. If it happens, it happened for a reason. It runs in my family, too, so I wouldn’t feel any type of way. E20: [Do you think you might get diabetes?] Yeah, probably. When it comes to me, I have this idea of where if it happens, it happens, so I don’t really think too much about it. E24: [If I get diabetes] well, then I’ve done it to myself, and I can’t take it back. So, if and when I got it, I do.	E15: I don’t think I’m gonna get it [diabetes] one day. I don’t think so. I don’t think so. …I just feel like I’m not gonna have it. I don’t know”

**Table 4.** Potential Intervention Elements Aligning With Identified Barriers

COM-B DomainIntervention Need		Interviewees’ Intervention Ideas to Address Identified Needs
Capability	The gap between knowledge and behaviors	Hands-on skills-building on specific topics of interest (e.g., cooking classes, gym equipment instruction) Drop-in opportunities to learn (e.g., coaches available at the park, able to teach exercise skills) Provide free gym memberships that include exercise classes
Opportunity	Healthy food prohibitively expensive, despite existing food programs	Provide navigation support to connect youth with existing food programs (e.g., SNAP, pantries) Augment with additional food supports (e.g., produce, vouchers) Provide education and motivation to support youth in maximizing the health value of their benefits
	Transportation and child care needs restrict the ability to access resources	Provide free transportation options (e.g., passes, gas vouchers) Provide child care during programming and/or facilitate peer-sharing of child care) Ensure resources/programs are near where young adults already are
	Immersion in unhealthy environments makes change challenging	Share social media/online resources with tips on how to be active within current daily life Take field trips to help participants see beyond their neighborhood (e.g., nearby outdoor spaces) Provide mental health care and coping strategies that equip participants to withstand negative external pressures Build positive social connections with peers and coaches (e.g., peer-sharing sessions, group classes, field trips) Include opportunities for family members (e.g., parents, partners, siblings) to participate Explicitly discuss neighborhood disparities and family dynamics to acknowledge reality and share strategies to thrive (e.g., peer sharing, others’ success stories, tips and tricks)
	Lack of control over time makes change overwhelming	Careful planning of intervention timing and modality to avoid exacerbating time demands Offer online and flexible options
Motivation	Learning about diabetes risk is a wake-up call that can be hard to sustain	Accountability check-ins with a coach or text message reminders Provide ongoing programming outside of clinic visits Make programming fun (enhance intrinsic motivation for change)
	Variable motivation to enact behavior change: 4 subgroups, each with distinct intervention needs	Provide concrete supports and tools as above for those who are already motivated to change Encourage self-efficacy for those who feel less confident in their ability to make changes Provide education that diabetes is avoidable, or to take diabetes risk more seriously, for those who do not recognize this already

SNAP indicates *Supplemental Nutrition Assistance Program*.

Note: Interviewees’ ideas for interventions were summarized and mapped to identified needs within domains of the Capability-Opportunity-Motivation-Behavior change framework (COM-B) by the research team.
