# Supplementary material for: A Qualitative Study Exploring Diabetes Prevention Intervention Needs and Ideas Among Young Adults at High Risk for Diabetes
Source: J Nutr Educ Behav. Author manuscript; Available in PMC 2026 Sep 14. (PMC13572042; doi:10.1016/j.jneb.2026.01.014)
Supplement: Supplementary file 1 [file NIHMS2206849-supplement-Supplementary_Material.docx]

**Supplementary Material**

**eTable 1. Eligibility questions**

| **Question** | **Response for eligibility** |
| --- | --- |
| Are you ages 18-26? | Yes |
| Have you ever had any lab tests that show you might be at risk for developing diabetes or has a health care provider ever told you that you might be at risk for diabetes in the future? | Yes |
| Have you ever been diagnosed with Diabetes (not prediabetes or high risk, but actual Diabetes)? | No |

**eTable 2. Semi-structured interview guide questions**

| **Section** | **Questions** |
| --- | --- |
| ***Lifestyle and knowledge background*** | We’ll start with some questions to help us better understand your day-to-day life   1. Tell me about a typical weekday in your life starting from the time that you wake up, what you do during the day, until the time you go to sleep. 2. Now tell me about a typical weekend day 3. (If not already described) What are your main activities as far as work and school? 4. Do you have any responsibilities to care for anyone in your family? Tell me more about what is involved. 5. Tell me more about the typical foods that you eat and beverages that you drink for breakfast, lunch, dinner, and snacks?    a. Who prepares the food?    b. Who purchases the food?    c. How much time do you spend each day preparing food? 6. Do you feel like you have a good understanding of what makes food healthy?    a. If yes, tell me what you think makes foods healthy or unhealthy?    b. If yes, where did you learn about this?    c. What about drinks? 7. Do you think the food and drink you personally eat on a usual day is mostly healthy? Why or why not?    a. What helps you to eat healthy?    b. What gets in the way of eating healthy food? 8. Do you feel like you have a good understanding of the kind of exercise people need to be healthy?    a. If yes, where did you learn about this? 9. Do you think you get the amount of exercise you need to be healthy? Why or why not?    a. What helps you to exercise?    b. What gets in the way of exercising? 10. What types of exercise do you do? 11. Have you ever made a change to your eating or exercise habits that lasted?     a. How did you do that? Did you get any support with that? |
| ***Opinions on challenges/ facilitators to lifestyle change*** | In this next section we will ask you for your opinions on things that make it easier or harder for people your age to eat healthy and exercise.   1. A lot of people observe that young adults find it hard to eat healthy. Why do you think that is?    a. What are the things that you think make it hard for people to eat healthy?    b. (If not already covered) What are the things that you think make it hard for people to buy healthy food?    c. [Prompt if necessary] Here are some examples of things that might make eating healthy harder. Do you think that any of these have an effect on you and people you know? (if yes…tell me more about that) Cost of healthy food, availability of places to buy healthy food, knowing how to cook healthy food, friends and family not eating healthy food, advertising from food and drink companies, high stress?    d. Is there anything else you can think of that makes it harder for people your age to eat healthy? 2. In your experience is there anything that helps people to eat more healthy?    a. (If not already covered) Can you think of anything that makes it easier to buy healthier food?    b. Do you have any experience with programs that give people food or money to buy food, like food banks or CalFresh;    c. Do you or people you know have experience with programs that teach you how to eat healthy food; support from friends or family to eat healthy? If yes...tell me more about how that experience went for you    d. Is there anything else you can think of that makes it easier for people your age to eat healthy? 3. Many young adults say it is hard to get enough exercise, what things do you think make it harder for people your age to exercise?    a. (Prompt only if necessary) Here are some examples of things that might make exercising harder, Do you think that any of these have an effect on you and people you know? (if yes…tell me more about that) No good places to exercise in your neighborhood, gym memberships are expensive, not feeling motivated, not having enough time to exercise, high stress    b. Is there anything else you can think of that makes it harder for people your age to exercise? 4. When it comes to getting enough exercise, is there anything that makes it hard for you and people you know to exercise?    a. (Prompt only if necessary) Here are some examples of things that might make exercising easier, do have experience using any of these? Having nice parks nearby, having friends who want to exercise together, joining a team, making gyms cost less, learning about new kinds of exercise    b. Is there anything else you can think of that makes it easier for people your age to exercise?    c. Do you or anyone you know in your age group use these things to make exercising easier? Why or why not? 5. Do you think people your age feel like they have control over the food they eat and exercise they do, or do they feel like they are powerless?    a. Do you feel like you are influenced by social media or marketing campaigns? How do you feel about that? What impact does that have on your food and exercise habits?    b. Do you think knowing more about other things that companies and the government do that affect whether healthy food and exercise are available, would make you feel more or less motivated to make changes in your own life? |
| ***Diabetes awareness*** | Now we will ask you some questions about a health condition called prediabetes.   1. Have you ever heard of prediabetes?    a. If yes, what is your understanding of what prediabetes is? 2. Have you ever been told you have prediabetes? 3. Is prediabetes something you have talked about with your doctor?    a. If no, have you ever talked about having a higher than normal risk of diabetes with your doctor?    b. If yes, How did that conversation go, how did it make you feel? Do you wish it had gone differently? Did it make you feel more Motivated or more discouraged? 4. Does anyone in your family have diabetes? 5. Do you ever think that you might get diabetes some day?    a. If not, why not?    b. If yes, What makes you think so?    c. If yes, What do you think or feel about the idea of getting diabetes some day? 6. Have you ever tried to make any changes to lower your chances of getting diabetes?    a. If yes, how is that going for you? |
| ***Intervention Ideas*** | There are some programs that can help people not get diabetes, but these are more helpful in older people, and less helpful in young adults. We are hoping to learn more from young adults like you about what kind of program you think would be the best in your age group. In the future we will be using some of this information to help us design new programs for young people.   1. Can you think of any class or program you participated in before that helped change your life for the better?    a. What was it about that program that worked well for you? 2. If you had to design a program to help prevent diabetes in your age group what would it look like? 3. Some programs have had meetings, classes, or coaching. Do you think any of these might be helpful? What would you want to learn? 4. If you or someone your age were going to participate in a program,    a. Do you think online, in person, phone/text, or something else, would be best for people your age, and why?    b. What time of day do you think works best for people your age?    c. How often do you think would be the most helpful and realistic for someone your age to participate in a program? (if no response, can prompt Every day, Every week, Every month, one time, or something else) 5. [Prompt if not mentioned in above responses] Some programs give people resources, like help with signing up for food stamps or free food. What would be the most helpful resource to be able to eat healthy? 6. [Prompt if not mentioned already] Other resources help people to exercise, like a gym membership, or free exercise classes. What would be the most helpful resource to be able to exercise? 7. We have heard from other young adults about challenges that get in the way of eating healthy and exercising, like stress, feeling unmotivated or depressed, or time pressures. What do you think a program could do that would be helpful to work through these challenges? 8. A lot of times when people try to make changes they do it for a day or a week and then stop. Do you have any ideas of what would help you to make changes that you would be able to continue with in your daily life and stick with it over time? 9. If there were unlimited money to help people your age live healthier lives and lower their risk of diabetes, what do you think people should do to help people your age achieve these goals? |

**eFigure 1. Participant Recruitment Flowchart**

^a^ Per institutional policy, primary care providers were given the opportunity to opt patients out of eligibility prior to contact.

^b^ Did not attend their interview and did not respond to rescheduling attempts.

**eTable 3.** EHR-derived patient characteristics of interviewed vs. not-interviewed participants

| **Patient characteristic** | **Interviewed**  **(n=24)** | **Not Interviewed**  **(n=204)** | ***p*** |
| --- | --- | --- | --- |
| Female | 63% (15) | 40% (82) | 0.04 ^a^ |
| Primary Language Spanish in EHR listing (vs. English) | 33% (8) | 24% (49) | 0.32 ^a^ |
| Length of time since last PCP visit (months) | 13 | 21 | 0.02 ^c^ |
| Mean age at time of EHR query (years) | 22 | 22 | 0.21 ^c^ |

^a^ Chi-Square test of independence.

^c^ Two-tailed t-test

Comparison of patient characteristics among interviewed versus eligible but not interviewed samples. These characteristics are derived from the EHR and in some cases (e.g. primary language) may differ from interviewees’ self-reported responses on the survey.

**eTable 4: Contact and consent outcomes by demographic group**

| **Gender/Preferred Language** | **Total contacted^a^**  **(*n*)** | **Total reached on any contact attempt**  **% (*n*)** | **Responded to first call**  **% (*n*)** | **Responded to first text message^b^**  **% (*n*)** | **Responded to subsequent calls/texts^c^**  **% (*n*)** | **Reached but declined participation**  **% (*n*)** | **Interested but screened out^d^**  **% (*n*)** | **Interview completed**  **% (*n*)** |
| --- | --- | --- | --- | --- | --- | --- | --- | --- |
| **Male/Spanish** | 41 | 73% (30) | 44% (18) | 15% (6) | 15% (6) | 34% (14) | 22% (9) | 5% (2) |
| **Male/English** | 90 | 43% (39) | 36% (32) | 7% (6) | 1% (1) | 27% (24) | 7% (6) | 8% (7) |
| **Female/Spanish** | 16 | 63% (9) | 56% (9) | 6% (1) | 0% (0) | 13% (2) | 6% (1) | 38% (6) |
| **Female/English** | 81 | 53% (43) | 40% (32) | 4% (3) | 10% (8) | 37% (30) | 1% (1) | 11% (9) |
| **Total** | 228 | 53% (121) | 40% (91) | 7% (16) | 7% (15) | 31% (70) | 7% (17) | 11% (24) |

^a^ Contact stopped once thematic saturation was reached for Female/Spanish, Female/English, and Male/English; all possible eligible patients were contacted for Male/Spanish.

^b^ Participants not reached by the first call but responded to the subsequent text.

^c^ Participants who did not pick up the first call or respond to the first text message but were reached on subsequent attempts.

^d^ Reasons to have screened out this stage include having turned 26 after the EHR query, or answering “no” to “Have you ever had any lab tests that show you might be at risk for developing diabetes or has a health care provider ever told you that you might be at risk for diabetes in the future?” per IRB stipulation of not disclosing a previously unknown diagnosis to patients in the course of research.
